# Understanding optimal cadence dynamics: a systematic analysis of the power-velocity relationship in track cyclists with increasing exercise intensity

**DOI:** 10.3389/fphys.2024.1343601

**Published:** 2024-04-05

**Authors:** Anna Katharina Dunst, Clemens Hesse, Olaf Ueberschär

**Affiliations:** ^1^ Institute for Applied Training Science, Department of Endurance Sports, Leipzig, Germany; ^2^ German Cycling Federation, Frankfurt, Germany; ^3^ Magdeburg-Stendal University of Applied Sciences, Department of Engineering and Industrial Design, Magdeburg, Germany; ^4^ Institute for Applied Training Science, Department of Biomechanics, Leipzig, Germany

**Keywords:** optimal pedaling rate, muscle fiber recruitment, power-velocity profiles, force-velocity profiles, exercise physiology, mathematical modelling, size principle

## Abstract

**Background:** This study aimed to investigate the changes in force-velocity (F/v) and power-velocity (P/v) relationships with increasing work rate up to maximal oxygen uptake and to assess the resulting alterations in optimal cadence, particularly at characteristic metabolic states.

**Methods:** Fourteen professional track cyclists (9 sprinters, 5 endurance athletes) performed submaximal incremental tests, high-intensity cycling trials, and maximal sprints at varied cadences (60, 90, 120 rpm) on an SRM bicycle ergometer. Linear and non-linear regression analyses were used to assess the relationship between heart rate, oxygen uptake (V.O_2_), blood lactate concentration and power output at each pedaling rate. Work rates linked to various cardiopulmonary and metabolic states, including lactate threshold (LT1), maximal fat combustion (FAT_max_), maximal lactate steady-state (MLSS) and maximal oxygen uptake (V.O_2max_), were determined using cadence-specific inverse functions. These data were used to calculate state-specific force-velocity (F/v) and power-velocity (P/v) profiles, from which state-specific optimal cadences were derived. Additionally, fatigue-free profiles were generated from sprint data to illustrate the entire F/v and P/v continuum.

**Results:** HR, V.O_2_ demonstrated linear relationships, while BLC exhibited an exponential relationship with work rate, influenced by cadence (*p* < 0.05, η^2^ ≥ 0.655). Optimal cadence increased sigmoidally across all parameters, ranging from 66.18 ± 3.00 rpm at LT1, 76.01 ± 3.36 rpm at FAT_max_, 82.24 ± 2.59 rpm at MLSS, culminating at 84.49 ± 2.66 rpm at V.O_2max_ (*p* < 0.01, η^2^ = 0.936). A fatigue-free optimal cadence of 135 ± 11 rpm was identified. Sprinters and endurance athletes showed no differences in optimal cadences, except for the fatigue-free optimum (*p* < 0.001, d = 2.215).

**Conclusion:** Optimal cadence increases sigmoidally with exercise intensity up to maximal aerobic power, irrespective of the athlete’s physical condition or discipline. Threshold-specific changes in optimal cadence suggest a shift in muscle fiber type recruitment toward faster types beyond these thresholds. Moreover, the results indicate the need to integrate movement velocity into Henneman’s hierarchical size principle and the critical power curve. Consequently, intensity zones should be presented as a function of movement velocity rather than in absolute terms.

## 1 Introduction

The velocity of movement is a critical determinant of various aspects of physical performance in sports. Cadence, measured in pedal revolutions per minute (rpm), is a key factor influencing metabolic responses during cycling across varying exercise intensities (Michaelis and Müller, 1942; Hess and Seusing, 1963 Eckermann and Millahn, 1967; Israel et al., 1967; Pugh, 1974; Gaesser and Brooks, 1975; McKay and Banister, 1976; Schürch et al., 1976; Seabury et al., 1977; Löllgen et al., 1980; Hagberg et al., 1981; Böning et al., 1984; Chavarren and Calbet, 1999; Zoladz et al., 2000; Foss and Hallén, 2004; Beneke and alkhatib, 2015; Beneke et al., 2018). At lower exercise intensities, a higher cadence is associated with elevated blood lactate concentration (BLC), increased cardiac, and respiratory measures (Gaesser and Brooks, 1975; Chavarren and Calbet, 1999; Zoladz et al., 2000). Simultaneously, cadence-dependent oxygen uptake- and carbon dioxide expiration kinetics tend to converge at maximal aerobic power (PV.O_2max_) (Zoladz et al., 2000). This leads to reduced performance at higher cadences (90–100 rpm) compared to lower cadences (40–60 rpm) across various submaximal physiological states, including BLC of 2 mmol L^-1^ and 4 mmol L^-1^ (Buchanan and Weltman, 1985; Zoladz et al., 2000; Beneke and Leithäuser, 2017), highlighting intensity-dependent cadence effects on gross efficiency (Hughes et al., 1982).

These cadence-dependent metabolic differences have been linked to specific muscle fiber activation patterns, particularly the premature recruitment of fast-twitch muscle fibers at higher cadences during lower exercise intensities (Macintosh et al., 2000; Sanderson et al., 2006). These patterns occur due to the distinct biomechanical and metabolic properties of muscle fiber types (I, IIa, IIx). These properties include contraction velocity, myosin ATPase activity, and the content of anaerobic glycolytic enzymes (Essén-Gustavsson and Henriksson, 1984; Sargeant, 1994), as well as aerobic mitochondrial protein content, defining the dominant metabolic pathways and thereby efficient contraction velocities (Sargeant, 1994).

The precise impact of cadence on optimal cycling performance remains debated, with preferences among professional and elite cyclists varying widely (Lucia et al., 2001; Vogt et al., 2008). Generally, the relationships between force, power, and pedaling rate in cycling mirror those observed within an isolated muscle, with established linear and parabolic relationships between maximal mean pedal force, pedaling rate, and maximal power output (McCartney et al., 1983; Beelen and Sargeant, 1991; Sargeant, 1994; Dorel et al., 2005; Gardner, 2007; Abbiss et al., 2009; Debraux et al., 2013; Dunst et al., 2022). These findings suggest the existence of an optimal pedaling rate (PR_opt_) to achieve maximal power output, potentially influenced by muscle fiber type distribution (Hautier et al., 1996; Hansen et al., 2002) and neuromuscular capacity (Dunst et al., 2022).

Parabolic functions have also been established to describe the relationship between metabolic or cardio-pulmonary state and cadence at a given work rate (Böning et al., 1984; Zoladz et al., 2000), indicating an inverted U-shaped relationship between power and velocity even at submaximal intensities. This allows the use inverse functions of the underlying linear force-velocity relationships to determine the maximal power output and its corresponding optimal cadence within a specific physical state. In the context of heart rate, ‘optimal’ refers to the cadence that is associated with the highest mechanical power output for a given cardiac load. To determine the cadence that results in the lowest oxygen consumption per unit of mechanical power output, it is necessary to consider the relationship between oxygen uptake, power output, and cadence. The most efficient relationship between mechanical power output and metabolic cost can be determined by considering oxygen uptake and lactate concentration. Riding at this cadence enables a cyclist to achieve a given mechanical power output at a minimum metabolic cost.

Zoladz et al. (2000) suggest an increase in optimal cadence to maximize power output with rising exercise intensity. This can be attributed to the successive recruitment of less energetically efficient faster-twitching muscle fibers with higher optimal contraction velocities (Sargeant, 1994; Sbriccoli et al., 2009). Recent studies also indicate that fatigue during maximal efforts results in a decrease in the optimal pedaling rate. This is mainly due to the progressive fatigue of fast-twitch IIx muscle fibers (Laube et al., 1990; Sargeant, 1994; Buttelli et al., 1996; Dunst et al., 2022). However, the systematic pattern of this relationship across submaximal to maximal intensities is still unknown. This study aims to analyze force-velocity and power-velocity relationships across different metabolic states to investigate the change in optimal cadence with increasing work rate up to maximal oxygen uptake and to examine the optimal pedaling rate at characteristic metabolic thresholds. Based on previous observations, we hypothesize a systematic increase in optimal cadence with rising work intensity, potentially indicating the recruitment of faster-twitching muscle fibers in the propulsive muscles during cycling.

This hypothesis combines Henneman’s size principle (Henneman and Mendell, 1981) with fiber type-specific force-velocity and power-velocity properties (Sargeant, 2007). This combination could expand the scope of recruitment requirements by incorporating force and velocity dimensions. We also presume universal threshold-specific optimal cadences representing different contributions of fiber types to power output, with the specific magnitude of threshold-specific power output depending on the individual power level. Understanding these relationships can provide valuable insights into the contributions of different fiber types to power output at various exercise intensities, ultimately allowing the individual optimization of cadence for a given duration of effort.

## 2 Materials and methods

### 2.1 Participants

Fourteen male professional track cyclists (9 sprinters, 5 endurance athletes, age: 19.5 ± 3.8 years, height: 1.86 ± 0.04 m, body weight: 83.69 ± 7.35 kg, body fat percentage: 11.65% ± 1.75%) were included in this study. In order to ensure adequate neuromuscular and metabolic performance, only those athletes were selected who had shown a close linear F/v profiles (*R* ^2^ > 0.95) in previous tests and who had demonstrated a consistently high performance over all races per day in a track cycling event at international championships. All athletes underwent current medical examinations confirming good health and the ability to perform exhausting exercises. Participants were requested to abstain from alcohol and strenuous exercise, and to maintain their normal nutritional habits for 24 h prior to the experimental session. All participants gave written informed consent to participate in this study. The study was approved by the Institute’s Ethics Committee (ER_2022.02.06_20) and was conducted in accordance with the Declaration of Helsinki.

### 2.2 Exercise protocol

Each participant completed a single day of laboratory testing, which included a submaximal incremental test, a ramp test to volitional exhaustion, and three series of exercise consisting of a 6-s maximal sprint followed by 4 min of high-intensity cycling. All tests were conducted in an isokinetic mode at a predetermined constant cadence. The incremental test, sprints, and 4-min tests were performed at three different pedaling rates (60, 90, and 120 rpm) to investigate the cadence-specific cardio-pulmonary and metabolic response (HR, V.O_2_, V.CO_2_, BLC). All tests were performed in a seated position on an SRM cycle ergometer (Schoberer Radmesstechnik GmbH, Jülich/Germany) with settings that reflected the actual individual competition conditions. Participants used their own cycling shoes and pedals. As the test protocol was similar to the daily training and competition of the track cyclists, who had previously performed similar performance testing in the laboratory, additional familiarization with the test was not considered necessary.

### 2.3 The incremental test

The incremental test consisted of work rates j) from 100 W to 300 W (j = {100; 140; 180; 220; 260; 300}) at pedaling rates i) of 60, 90, and 120 rpm (i = {60; 90; 120}). Starting with a work rate of 100 W, participants exercised for 9 min at each work rate, changing the cadence from 60 to 90–120 rpm every 3 min. The work rate was then increased by 40 W until a blood lactate concentration greater than 4 mmol L^-1^ or a respiratory exchange ratio consistently ≥1.0 was reached during the stage. The sequence of cadence and work rate combinations was arranged based on the results of preliminary experiments to ensure that participants reached a steady state at each stage and cadence during the 3 min. Validation of the steady state condition was confirmed by observing a plateau in both V.O_2_ and heart rate kinetics following an exponential increase, consistent with the description of Xu and Rhodes (1999).

### 2.4 The ramp test

After the incremental test, participants rested passively for at least 10 min to ensure sufficient energy availability to reach peak oxygen uptake during the subsequent ramp test. The next test began when the participant’s blood levels of lactate and glucose had returned to the pre-test values. During the ramp test, power was increased by 10 W every 10 s from a baseline of 140 W to ensure that voluntary exhaustion was reached within <10 min (Yoon et al., 2007). The test was performed at 90 rpm and ended when the cadence fell below 60 rpm despite maximal voluntary effort.

### 2.5 6-s sprints and 4-min high-intensity cycling

Three hours after completing the ramp test, participants performed three sets of exercise. Each set consisted of a 6-s maximal cycling sprint followed by 4 min of high-intensity cycling (≥90% PV.O_2max_), maintaining a constant pedaling rate of 60, 90, and 120 rpm throughout the sessions. To avoid potential confounding effects, such as varying levels of fatigue or post-activation performance enhancement, the order of the pedaling rates was randomized. A 15-min period of passive rest was implemented between each exercise set to reduce the potential carryover of fatigue.

The warm-up before each sprint consisted of 6 min of low-intensity cycling at 1–1.5 W kg^-1^ of body weight, followed by a 3-s maximal sprint. Each athlete rested passively for 10 min between warm-up and testing. During the subsequent sprints, participants were instructed to achieve the target cadence as quickly as possible and then exert maximal effort throughout the 6-s duration, with continuous verbal encouragement provided. A 10-min passive rest interval separated the 6-s sprints from the subsequent 4-min high-intensity cycling bout.

For the 4-min high-intensity cycling test, cadence-specific maximal aerobic power was calculated as the work rate corresponding to the highest oxygen uptake observed during the ramp test, using the slope ( $C_{E_{\dot{V}O2i}}$ ) and baseline level (V.O_2iBase_) of the cadence-specific V.O_2_-P relationship^1^ (PV.O_2max_(PR_i_)=(V.O_2max_–V.O_2iBase_)·C_EV.__O2i_^−1^), following the procedure outlined by Joyner and Coyle (2008). This approach ensured to match the work rate for the high-intensity cycling test to the individual’s physiological capacity at the respective pedaling rate.

### 2.6 Data collection and measurements

During the exercise protocol, an SRM power meter continuously monitored the crank torque M) and angular velocity ω) at an internal sampling rate of 500 Hz. The data was sampled at a rate of 10 Hz and consisted of power and pedaling rate measurements. The average tangential force F) at both pedals during a single revolution, accounting for individual crank length, was calculated. Heart rate and respiratory data were measured continuously using a heart rate monitor (Polar, Polar Electro Oy, Kempele/Finland) and a breath-by-breath portable gas analyzer (Metamax 3B, Cortex, Leipzig/Germany) from 2 min before the beginning of the warm-up to measure resting heart rate and oxygen uptake up to the fourth minute post-exercise. The gas analyzer was calibrated before each test using a known concentration of gases and a 3-L syringe following the manufacturer’s recommendations. Blood lactate concentration was measured immediately before and after exercise, as well as 1, 3, 5, 7, and 10 min after each test, by collecting 20 µL capillary blood from the hyperemic earlobe for hemolysis and enzymatic-amperometric determination of lactate (Biosen, EKF Diagnostics, Magdeburg/Germany). Lactic acid concentration was also measured during the final 15 s of each stage of the incremental test.

### 2.7 Data processing

Steady-state measurements of heart rate (HR_ij_), oxygen uptake (V.O_2ij_), and respiratory exchange ratio (RER_ij_) were determined as the average values over the last 30 s of each trial for each pedaling rate i at work rate j within the specified ranges. Linear and non-linear regression analyses were performed for each pedaling rate separately to investigate the cadence-specific physiological response across increasing work rates. The relationships between heart rate and power output (HR_i_(P)) and between oxygen uptake and power output (V.O_2i_(P)) were analyzed using linear regression analysis based on the steady-state data obtained during the incremental test. The equations used were:

$${\text{HR}}_{i}\left(P\right)=C_{{\text{E}}_{\text{HRi}}}\cdot P+{\text{HR}}_{\text{iBase}}$$ (4.1)

$$\dot{V}O_{2i}\left(P\right)=C_{E_{\dot{V}O2i}}\cdot P+\dot{V}O_{2\text{iBase}}$$ (4.2)

In these equations, the slope of the linear functions represents the efficiency constants for heart rate ( $C_{{\text{E}}_{\text{HRi}}}$ [bpm W^−1^]) and oxygen uptake ( $C_{E_{\dot{V}O2i}}$ [ml min^−1^ W^−1^]), while the *y*-axis intercept denotes their corresponding baseline levels (V.O_2iBase_ [ml min^−1^], HR_iBase_ [bpm]). Work rate-specific metabolic costs (W_MET_) were determined via indirect calorimetry from V.O_2_, employing a caloric equivalent determined by the mean respiratory exchange ratio (di Prampero, 1981; Ueberschär et al., 2019).

Non-linear regression analysis was conducted using a mono-exponential function to analyze pedaling rate-specific blood lactate concentrations as a function of the work rate (Hughson et al., 1987). The BLC data were derived from both the incremental test and the 4-min test. The function used was:

$${\text{BLC}}_{i}\left(P\right)=A_{i}\cdot e^{P\cdot \tau i}+C_{i},$$ (4.3)

with cadence specific amplitude A_i_ (mmol L^-1^), time constant τ_i_ (mmol L^-1^ W^−1^) and baseline level C_i_ (mmol L^-1^).

Then, the inverse functions HR_i_^−1^(P), V.O_2i_^−1^(P), and BLC_i_^-1^(P) were calculated as follows:

$$P\left({\text{HR}}_{i}\right)=\frac{{\text{HR}}_{i}‐{\text{HR}}_{i\text{Base}}}{C_{E_{\text{HRi}}}}$$ (5.1)

$$P\left(\dot{V}O_{2i}\right)=\frac{\dot{V}O_{2i}‐\dot{V}O_{2\text{iBase}}}{C_{E_{\dot{V}O_{2i}}}}$$ (5.2)

$$P\left({\text{BLC}}_{i}\right)=\frac{\ln\text{(}\frac{{\text{BLC}}_{i}‐C_{i}}{A_{i}}\text{)}}{\tau_{i}}$$ (5.3)

These functions were used to compute power outputs and the underlying pedal forces (F(v) = P(v)·v^−1^) corresponding to specific cardiopulmonary and metabolic states across the three pedaling rates, forming the basis for state-specific force-velocity (F/v) and power-velocity (P/v) profiles.

Linear and non-linear regression analyses were performed to derive 1) intensity- and threshold-specific and 2) fatigue-free F/v and P/v profiles. The calculations were based on 1) data extracted from equations (5) and 2) data obtained during the first 3 s of each maximal sprint (Dunst et al., 2022; Dunst et al., 2023a). In each case, the following linear function:

$$F\left(v\right)=a∙\mathrm{PR}+b,$$ (6)

approximates the relationship between mean pedal force F (N) and cadence PR (rpm) as specific movement velocity. The relationship between power and cadence can be described mathematically using a second-order polynomial function P(v), multiplying F(v) by PR. These functions enabled the calculation of important performance indices, such as the maximal mean pedal force (F_max_ = b [N]), maximal cadence (PR_max_ = -b·a^−1^ [rpm]), maximal power output (P_max_ = -b^2^·(4a)^−1^ [W]), and the corresponding optimal cadence (PR_opt_ = 0.5·PR_max_ [rpm]), for each specific submaximal and maximal state. The validity criterion for the profile was *R* ^2^ > 0.95. In the case of fatigue-free profiles, the condition P_max_ ≥ the highest measured power (P_peak_ [W]) must also be met.

The data points calculated for intensity-specific optimal cadences were analyzed to identify any systematic changes in optimal cadence with increasing intensity, ranging from very low (near baseline) to maximal aerobic effort. After visually inspecting the observed patterns, non-linear regression techniques were used, employing sigmoidal model functions for this purpose.

To calculate cadence-specific power output corresponding to different metabolic thresholds and maximal aerobic effort, the maximal oxygen uptake and maximal lactate accumulation rate (v.La_max_) were determined (Mader and Heck, 1994, Mader, 2003). The maximal 10-s mean oxygen uptake during the ramp test was used to determine the test-specific V.O_2max_. Exhaustion was assumed, when two or more of the following criteria were met: respiratory exchange ratio exceeded 1.1, blood lactate concentration exceeded 8 mmol L^-1^, or the minute ventilation to oxygen uptake ratio (V.E·V.O2^−1^) surpassed 30. The v.La_max_ was determined based on the highest blood lactate accumulation during the sprint tests as described in our recent publication (Dunst et al., 2023a). The post-exercise blood lactate concentration was analyzed using non-linear regression with the 3-parameter model recommended by Beneke et al. (2005) to calculate the post-exercise maximum (BLC_max_).

Metabolic thresholds, including lactate threshold 1 (LT1: the highest work rate without significant blood lactate accumulation), the work rate associated with peak fat oxidation (FAT_max_: synonymous with Maders’ maximum pyruvate deficit), and maximal lactate steady state (MLSS: crossing point at which lactate formation equals exactly the maximal rate of lactate elimination via oxidative phosphorylation), were determined based on V.O_2max_ and v.La_max_, following the methodology of Mader and Heck (1996), and Mader (2003), explicitly described in the Supplementary Material. The cadence-specific power output corresponding to these thresholds, as well as to the maximal oxygen uptake, was calculated using the cadence-specific individual oxygen demand per Watt ( $C_{E_{\dot{V}O2i}}$ ).

### 2.8 Statistical analyses

The data were checked for normality using the Shapiro-Wilk test and are presented as means ± SD. A two-factorial analysis of variance, along with non-linear regression calculations, was employed for statistical assessment, considering discipline as a within-factor, with the three different pedaling rates as independent factors. The effect sizes are reported as partial eta-squared (η^2^), where ≥0.01, ≥0.06, ≥0.14 indicate small, moderate, and large effects, respectively (Cohen, 1988). Bonferroni *post hoc* tests were performed to determine where significant differences occurred, when appropriate. Post-hoc t-tests for independent samples were used to analyze the mean differences between the parameters between sprint and endurance athletes. For pairwise effect size comparison, standardized mean differences (SMD) are also calculated (trivial: d < 0.2, small: 0.2 ≤ d < 0.5, moderate: 0.5 ≤ d < 0.8, large: d ≥ 0.8) (Cohen, 1988). Statistical significance was set at an alpha level of <0.05. The quality of the regression analyses was examined by calculating the coefficient of determination *R* ^2^. Mathematical analysis and statistical tests were processed using IBM SPSS statistics version 24 Software for Windows (SPSS Inc., Chicago, IL, United States), Office Excel 2016 (Microsoft Corporation, Redmond, WA, United States), and MATLAB 9.10.0 R2021a (The MathWorks, Inc., Natick, MA).

## 3 Results

In accordance with their individual performance level, all athletes successfully completed the 220 W stage. Thirteen athletes progressed to the 260 W stage, and six athletes completed the 300 W stage of the incremental test. Within each stage, significant differences were observed for each parameter (HR, V.O_2_, BLC) across the three pedaling rates, as detailed in Table 1. The analysis of the differences in physiological responses during the incremental test between sprinters and endurance athletes was based on the coefficients of the model functions HR(P), V.O_2_(P) and BLC(P).

**TABLE 1:** Mean values of heart rate (HR), oxygen uptake (VO_2_), and blood lactate concentration (BLC) across the different stages of the incremental test for the three pedaling rates (60, 90, 120 rpm). The number of athletes completing each stage varied due to individual differences in performance: for 220 W, n = 14; for 260 W, n = 12; and for 300 W, n = 6.

Parameter	Stage	60 rpm		90 rpm		120 rpm		ANOVA	
		M	SD	M	SD	M	SD	*p*	η^2^
HR (bpm)	100 W	105.60	12.40	114.36	12.47	131.72	12.39	.000	.962
	140 W	122.34	12.57	131.18	13.45	144.55	11.18	.000	.963
	180 W	137.43	12.70	145.96	13.33	158.82	11.81	.000	.942
	220 W	152.83	13.54	160.28	13.00	172.08	10.86	.000	.913
	260 W	168.17	15.31	174.42	14.25	182.87	11.49	.000	.872
	300 W	176.06	12.71	183.71	10.80	188.22	12.20	.000	.929
V.O_2_ (L min^−1^)	100 W	0.82	0.25	0.88	0.28	1.20	0.39	.000	.957
	140 W	1.06	0.41	1.07	0.45	1.49	0.54	.000	.956
	180 W	1.25	0.58	1.49	0.67	2.02	0.87	.000	.962
	220 W	1.90	0.84	2.33	1.16	3.09	1.29	.000	.955
	260 W	2.59	0.80	2.89	0.97	4.07	1.17	.000	.896
	300 W	3.94	0.16	4.12	0.18	4.64	0.25	.000	.969
BLC (mmol L^−1^)	100 W	0.82	0.25	0.88	0.28	1.20	0.39	.000	.763
	140 W	1.06	0.41	1.07	0.45	1.49	0.54	.000	.766
	180 W	1.25	0.58	1.49	0.67	2.02	0.87	.000	.804
	220 W	1.90	0.84	2.33	1.16	3.09	1.29	.000	.593
	260 W	2.59	0.80	2.89	0.97	4.07	1.17	.000	.714
	300 W	2.89	0.97	3.24	1.08	4.51	1.28	.007	.635

### 3.1 Cardiopulmonary and metabolic response during the incremental test at different pedaling rates

**FIGURE 1:**
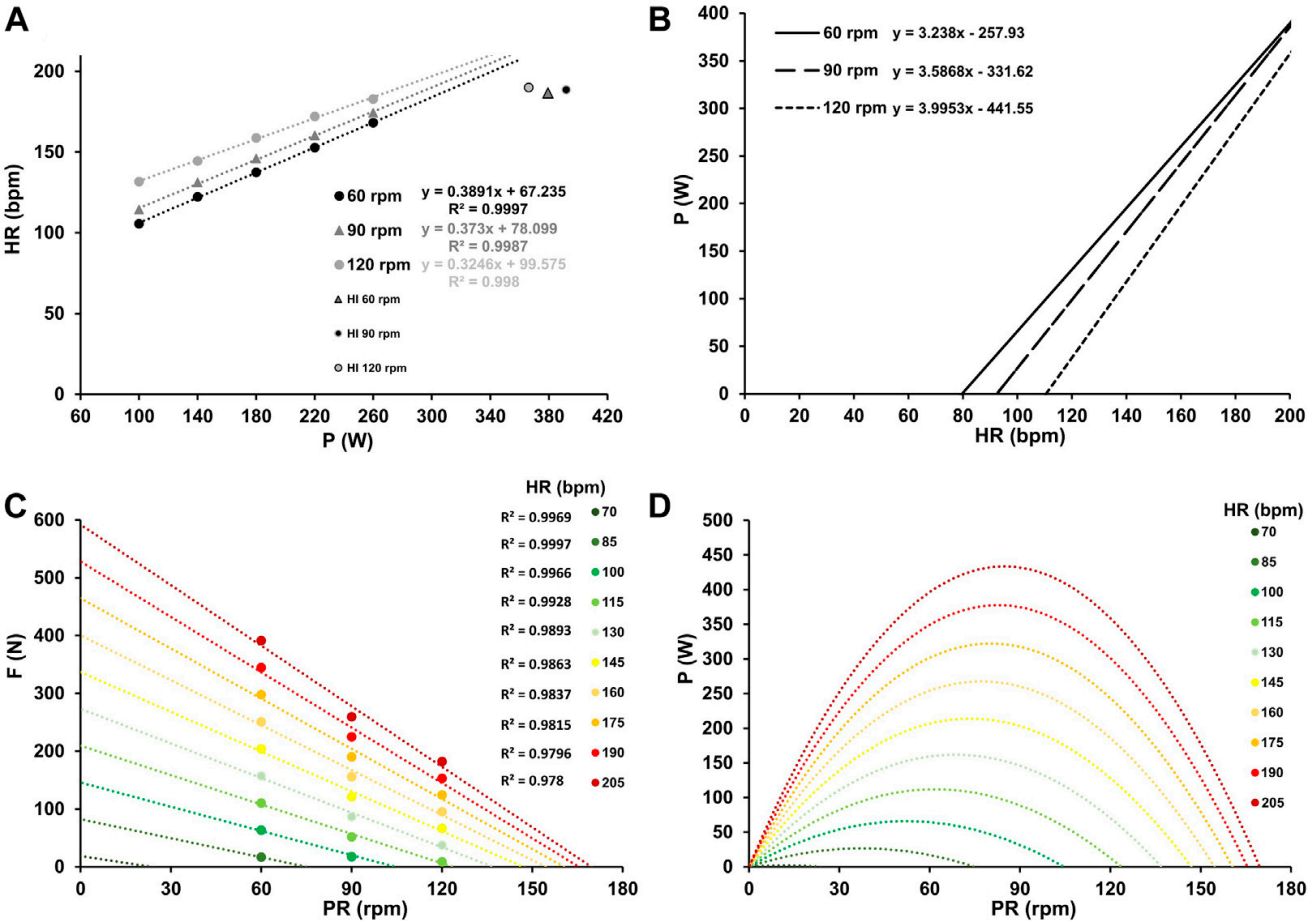
Mean heart rate response at work rates ranging from 100–260 W for the three different pedaling rates with the corresponding linear relationship between heart rate and work rate, along with their respective coefficients of determination **(A)**. Inverse relationship between power output and heart rate for the three different pedaling rates **(B)**. Force-velocity profiles **(C)** and power-velocity profiles **(D)** at various heart rates, ranging from low (70 bpm) to maximum (205 bpm). In Figure A, the values at HI were considered as outliers due to the high discrepancy between prediction and actual heart rate at HI. Their exclusion from the analysis did not affect the model of optimal cadence with increasing heart rate.

**FIGURE 2:**
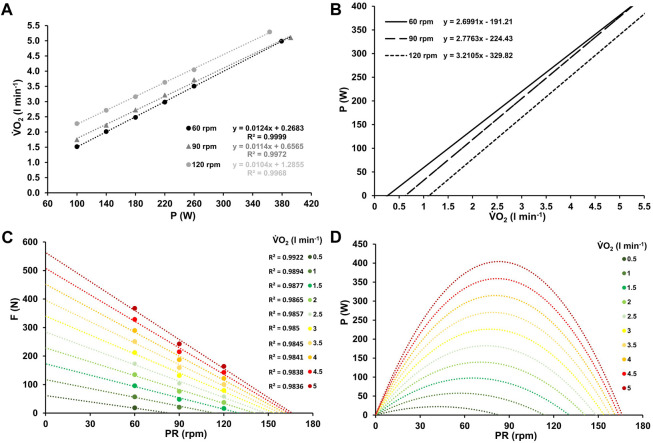
Mean oxygen uptake at work rates ranging from 100–260 W and at high-intensity (≥90% PV.O_2max_) for the three different pedaling rates with the corresponding linear relationship between oxygen uptake and work rate, along with their respective coefficients of determination **(A)**. Inverse relationship between power output and oxygen uptake for the three different pedaling rates **(B)**. Force-velocity profiles **(C)** and power-velocity profiles **(D)** at various levels of oxygen uptake, ranging from low (0.5 L min^-1^) to maximum (5 L min^-1^).

Heart rate and oxygen uptake increased linearly with power output at each pedalling rate (see Figure 1A; Figure 2A). Table 2 summarizes the regression equations for heart rate (HR) and oxygen uptake (V.O_2_), along with the corresponding coefficients of determination (*R* ^2^).

**TABLE 2:** Regression equation for heart rate (HR [bpm] = C_EHRi_ [bpm·W^−1^]·x [W]+HR_Base_ [bpm]) and oxygen uptake (V.O_2_ [ml min^-1^] = C_EVO2i_ [ml·min^-1^·W^−1^]·x [W]+VO_2Base_ [ml·min^-1^]) with coefficients of determination (*R* ^2^).

		C_E_		Base		*R* ^2^	
Parameter	PR	M	SD	M	SD	M	SD
HR (bpm)	60 rpm	0.39	0.08	69.10	16.86	0.995	0.003
	90 rpm	0.37	0.06	79.91	16.74	0.994	0.004
	120 rpm	0.32	0.06	101.59	17.42	0.996	0.005
V.O_2_ (ml min^−1^)	60 rpm	12.43	0.59	274.11	138.13	0.996	0.003
	90 rpm	11.66	0.89	624.82	6.13	0.994	0.006
	120 rpm	11.27	0.80	1,155.98	216.89	0.992	0.010

**FIGURE 3:**
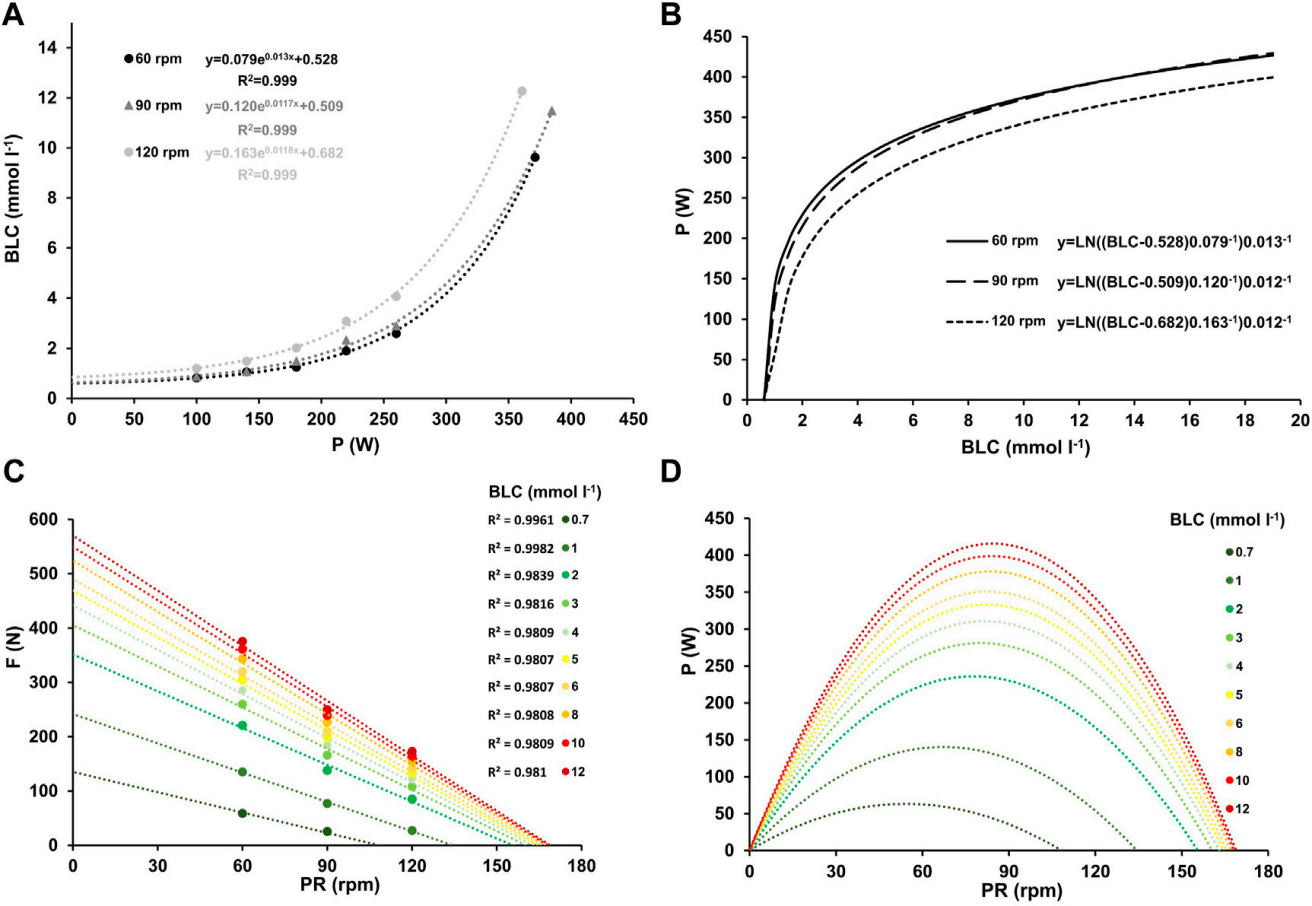
Mean blood lactate concentration at work rates ranging from 100–260 W and at high-intensity (≥90% PV.O_2max_) for the three different pedaling rates with the corresponding linear relationship between blood lactate concentration and work rate, along with their respective coefficients of determination **(A)**. Inverse relationship between power output and blood lactate concentration for the three different pedaling rates **(B)**. Force-velocity profiles **(C)** and power-velocity profiles **(D)** at various blood lactate concentrations, ranging from low (0.7 mmol L^-1^) to maximum (12 mmol L^-1^).

Blood lactate concentration increased exponentially with power output at each cadence, as shown in Figure 3A. Table 3 presents the regression equations for blood lactate concentration (BLC) and the corresponding coefficients of determination.

**TABLE 3:** Regression equation for blood lactate concentration (BLC [mmol·L^-1^] = A [mmol·L^-1^]· ex[W]·τ [mmol L-1W-1] +C [mmol·L^-1^]) with coefficients of determination (*R* ^2^).

		A		τ		C		*R* ^2^	
Parameter	PR	M	SD	M	SD	M	SD	M	SD
BLC (mmol L^−1^)	60 rpm	0.273	0.184	0.0100	0.0025	0.150	0.217	0.979	0.017
	90 rpm	0.268	0.170	0.0102	0.0024	0.110	0.170	0.982	0.014
	120 rpm	0.378	0.244	0.0100	0.0023	0.208	0.228	0.985	0.011

The slopes of the HR-P and V.O_2_-P relationships during incremental cycling showed significant differences between pedaling rates (F = 30.131, *p* < 0.001, η^2^ = 0.733; F = 12.144, *p* < 0.001, η^2^ = 0.503). Baseline HR and V.O_2_ levels, determined from the *y*-axis intercept of the HR-P and V.O_2_-P relationships during incremental cycling, were significantly higher at higher pedaling rates (F = 156.590, *p* < 0.001, η^2^ = 0.934; F = 144.423, *p* < 0.001, η^2^ = 0.923). While the amplitude and time constant of the BLC-P relationship did not demonstrate significant differences between cadences (*p* > 0.05), baseline values were significantly higher at 120 rpm (F = 6.856, *p* = 0.004, η^2^ = 0.364).

Figures 1, 2, and 3A the mean heart rate, mean oxygen uptake, and mean blood lactate concentration at work rates ranging from 100 to 260 W, and at high-intensity (HI), along with the corresponding linear or exponential relationship for three different pedaling rates. The graphs (B) present the inverse functions of work rate for heart rate, oxygen uptake, and blood lactate concentration, along with their respective coefficients of determination (*R* ^2^). The force-velocity (C) and power-velocity profiles (D) were calculated for different cardiopulmonary and metabolic states using these inverse functions. The quality of the regression lines is reported in Figure 1C, Figure 2C, and Figure 3C.

**FIGURE 4:**
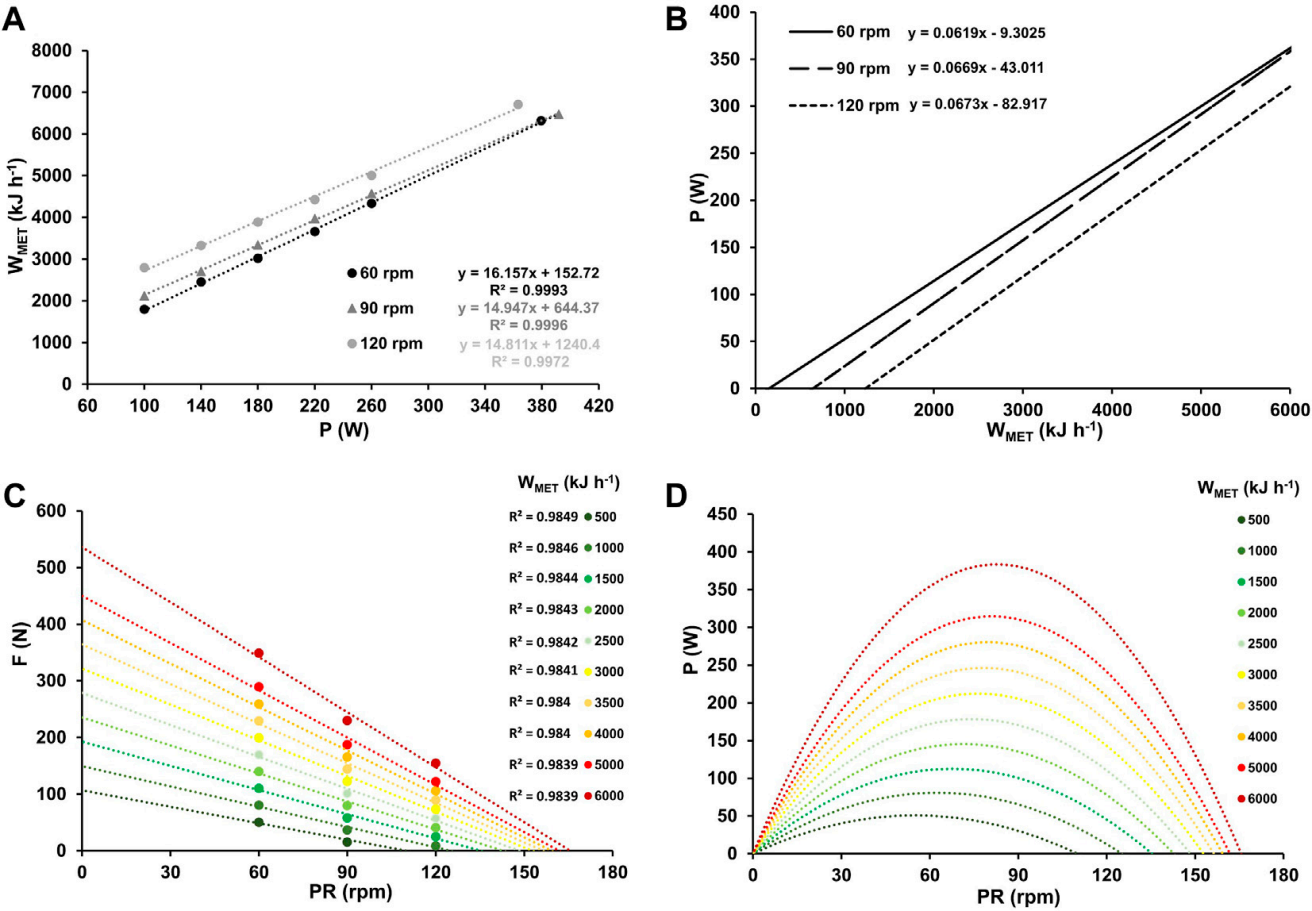
Mean metabolic work rates (W_MET_) at work rates ranging from 100–260 W and at high-intensity (≥90% PV.O_2max_) for the three different pedaling rates with the corresponding linear relationship between W_MET_ and work rate, along with their respective coefficients of determination **(A)**. Inverse relationship between power output and metabolic work rate for the three different pedaling rates **(B)**. Force-velocity profiles **(C)** and power-velocity profiles **(D)** at various W_MET_ values, ranging from low (500 kJ h^-1^) to maximum (6,000 kJ h^-1^).

Figures 4A–D display work rate-specific metabolic costs for three different pedaling rates, along with the corresponding inverse relationship between power output and metabolic work rate. The figure also shows force-velocity profiles and power-velocity profiles at various W_MET_ values, ranging from low to maximal aerobic work intensity.

### 3.2 Optimal pedaling rate at different cardiopulmonary and metabolic states

**FIGURE 5:**
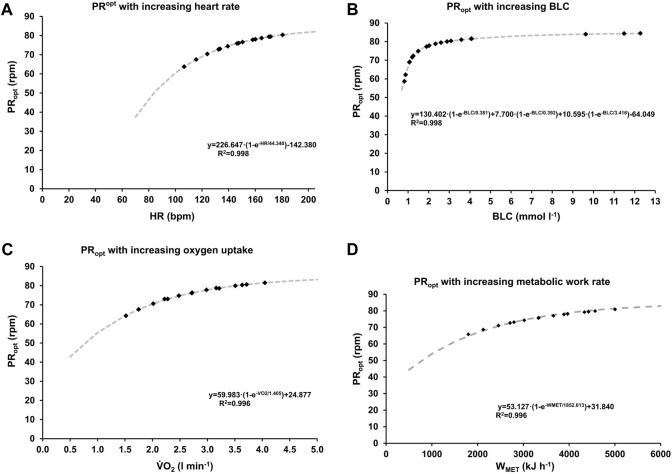
Optimal pedaling rate as a function of heart rate **(A)**, oxygen uptake **(B)**, blood lactate concentration **(C)** and metabolic work rate **(D)**. The grey dashed lines represent functions derived from non-linear regression analysis of the data depicted in Figures 1–4. The data points, represented as squares, illustrate the mean values across various stages and pedaling rates.

The optimal pedaling rate for different cardiopulmonary and metabolic states was determined using state-specific F/v and P/v profiles. Non-linear regression analysis revealed a sigmoidal relationship between work rate and optimal pedaling rate for HR and V.O_2_, while BLC exhibited a tri-component sigmoidal relationship. The derived functions for HR, V.O_2_, and BLC exhibited high goodness of fit (*R* ^2^ > 0.996). The model functions depicting optimal pedaling rate in relation to increasing heart rate, oxygen uptake, and blood lactate concentration are presented in Figure 5. Across all parameters, optimal pedaling rate exhibited a consistent exponential rise with intensity, ranging from approximately 45.90 ± 2.24 rpm (HR), 45.49 ± 1.89 rpm (V.O_2_), and 45.31 ± 1.38 rpm (BLC) at individual minimal intensity (80 ± 18 bpm, 511 ± 170 mL, 0.5 ± 0.2 mmol L^-1^) to an average of approximately 86.43 ± 2.65 rpm (HR), 85.81 ± 2.73 rpm (V.O_2_), and 84.67 ± 2.29 rpm (BLC) at maximum, without any statistically significant differences between parameters.

**FIGURE 6:**
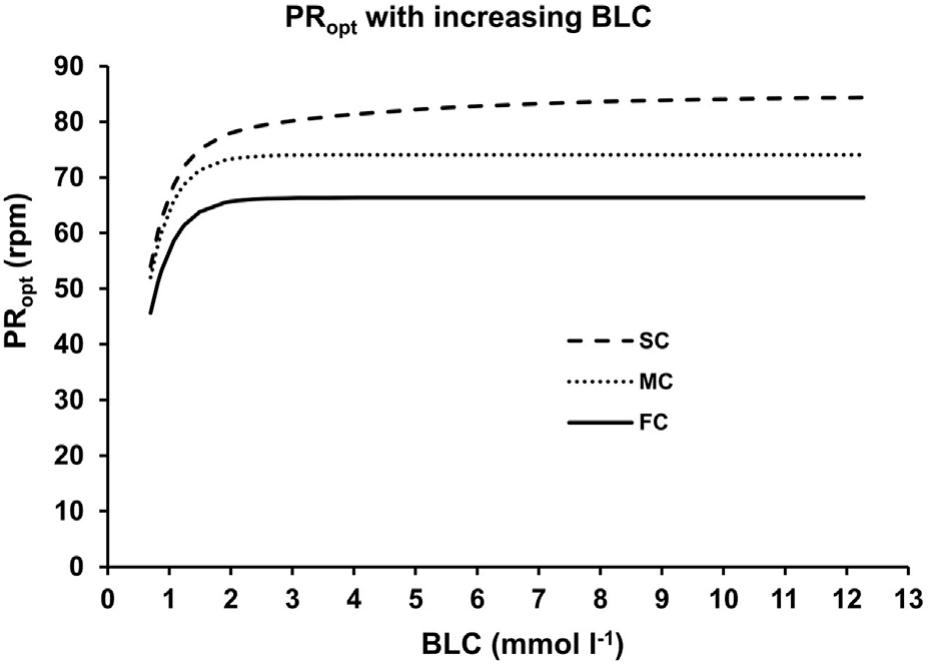
The three different components of the optimal pedaling rate as a function of blood lactate concentration. The lines represent the fast (FC), medium (MC) and slow (SC) components derived from non-linear regression analysis of the data depicted in Figure 4.

A thorough examination of the optimal cadence as influenced by blood lactate concentration unveiled three statistically significant components (FC: fast component, MC: medium component, SC: slow component) across all athletes, as displayed in Figure 6. The limit values for these PR_opt_(BLC)-components were 66.22 ± 4.96 rpm, 75.29 ± 2.83 rpm, and 84.86 ± 2.39 rpm, while their associated time constants were 0.36 ± 0.18 rpm (mmol L^-1^)^−1^, 0.39 ± 0.18 rpm (mmol L^-1^)^−1^, and 4.21 ± 1.72 rpm (mmol L^-1^)^−1^. These values exhibited notable statistical distinctions (F ≥ 47.483, *p* < 0.001, η^2^ ≥ 0.798). No statistically significant differences between sprinters and endurance athletes could be observed (*p* > 0.05).

### 3.3 Force-velocity and power-velocity profiles at different metabolic thresholds

Test-specific maximal oxygen uptake was found to average 62.6 ± 9.4 mL kg^-1^ min^-1^, with a maximal blood lactate accumulation rate of 0.80 ± 0.20 mmol L^-1^ s^-1^. Power outputs at LT1, FAT_max_, MLSS, and V.O_2max_ were 110 ± 30 W, 175 ± 49 W, 278 ± 54 W, and 392 ± 45 W, respectively, at a pedaling rate of 90 rpm. This cadence is commonly used in cycling performance diagnostics.

The fatigue-free F/v profile revealed a mean maximal force of 1,282 ± 188 N and an average calculated maximal crank velocity of 270 ± 21 rpm, resulting in a peak power output of 1,523 ± 282 W at the corresponding optimal pedaling rate of 135 ± 11 rpm. The slope of this profile was a = −4.74 ± 0.57 N rpm^-1^, with an *R* ^2^ exceeding 0.990 for all athletes. Significant differences were observed between sprinters and endurance athletes in terms of F_max_ (*p* < 0.001; d = 3.092), P_max_ (*p* < 0.001; d = 3.342), PR_max_ (*p* < 0.001; d = 2.215), V.O_2max_ (*p* < 0.001; d = −1.997), and power output at metabolic thresholds (*p* < 0.01; d ≤ −2.162).

The optimal pedaling rate at different metabolic thresholds, including LT1, FAT_max_, MLSS, and PV.O_2max_, was determined based on the submaximal F/v and P/v profiles. The mean optimal pedaling rates for these thresholds were 66.18 ± 3.00 rpm, 76.01 ± 3.36 rpm, 82.24 ± 2.59 rpm, and 84.49 ± 2.66 rpm, respectively. A statistically significant increase in optimal cadence was observed with the intensity of the corresponding metabolic threshold (F = 176.039, *p* < 0.01, η^2^ = 0.936).

**FIGURE 7:**
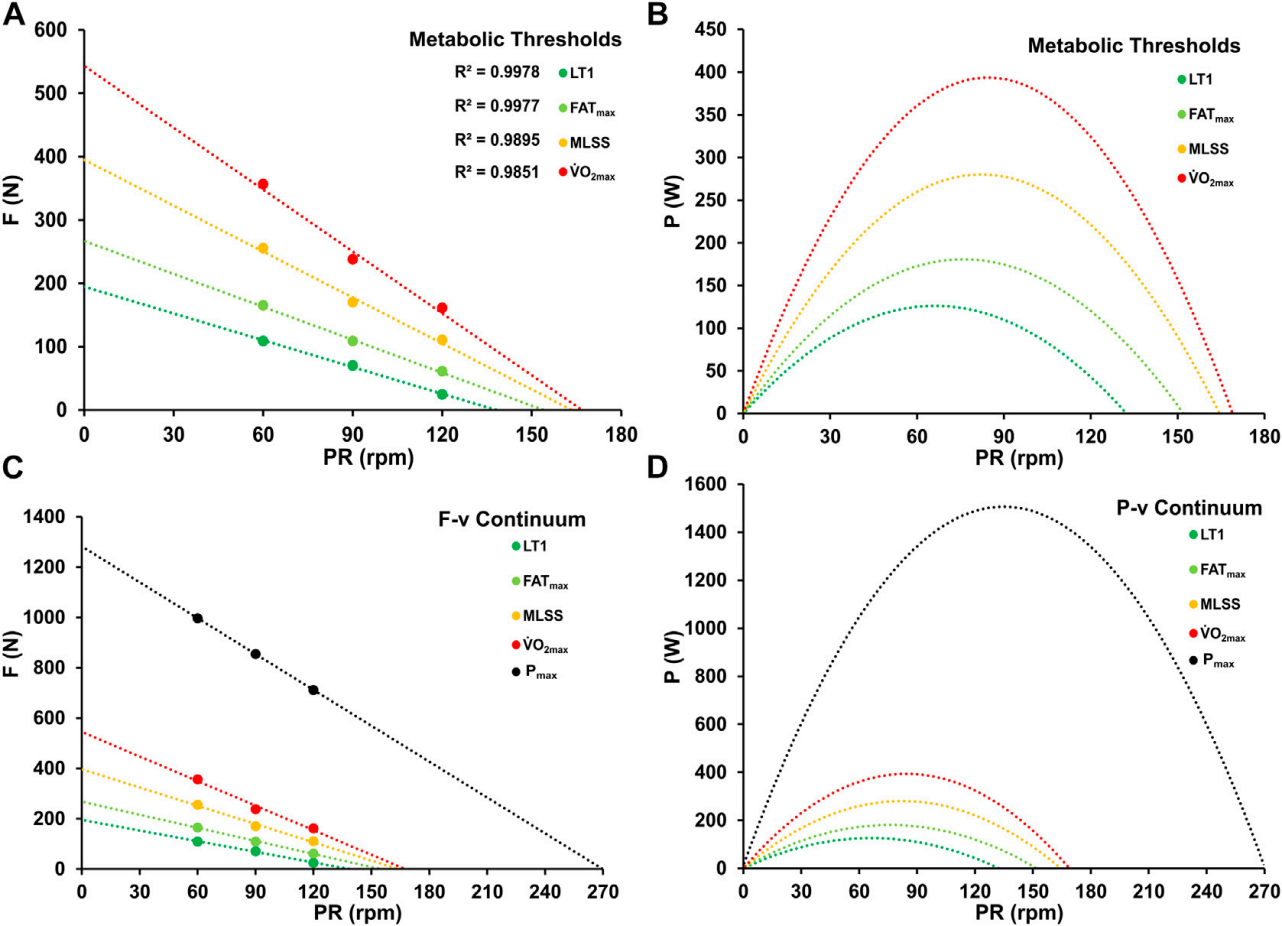
Force-velocity **(A, C)** and power-velocity profiles **(B, D)** at various metabolic thresholds (LT1, FAT_max_, MLSS, PV.O_2max_) and at maximal power output.

Comparing the maximal optimal pedaling rates derived from the different components of the PR_opt_-BLC relationship (FC, MC, SC) with the optima at LT1, FAT_max_, and PV.O_2max_, no significant differences were observed (*p* > 0.05). Likewise, no significant differences were observed in the asymptotic values of the three components FC, MC, SC or in optimal pedaling rates at metabolic thresholds between the groups (*p* > 0.05). Figure 7 shows the mean F/v and P/v profiles calculated for the different metabolic thresholds along with the fatigue-free maxima.

## 4 Discussion

The aim of this study was to determine the alteration in optimal cadence with increasing work rate up to maximal aerobic effort, by using force-velocity and power-velocity relationships across different cardiopulmonary and metabolic states, to analyze these changes for possible systematic patterns, and to examine the optimal cadence at characteristic metabolic thresholds. Our results indicate that the optimal cadence increases systematically and consistently with intensity, ranging from 45 rpm at very low intensity to 85 rpm at maximal aerobic power output, regardless of the specific metabolic parameter analyzed. At characteristic metabolic states, a significant increase in optimal cadence was observed, with PR_opt_ increasing from 65 rpm at LT1 to 85 rpm at V.O_2max_, without significant inter-individual differences. A detailed analysis of the pattern of changes in PR_opt_ with increasing blood lactate concentration suggests the possible existence of three distinct components (fast, medium, and slow components) characterized by significantly different activation constants and upper limits. The upper limits may represent the respective optimal cadences up to which a fiber type can contribute to mechanical power output, while the different time or activation constants may represent the metabolic state from which the respective fiber type is recruited. These findings suggest that adjusting the cadence for the power output and/or metabolic state could optimize cycling performance.

### 4.1 Cardiopulmonary and metabolic response during the incremental test at different pedaling rates

This study investigated the cardiopulmonary and metabolic responses during incremental cycling tests at different pedaling rates to derive state-specific F/v and P/v profiles. Consistent with previous findings, oxygen uptake and heart rate increased linearly with increasing work rate, regardless of the pedaling rate, and converged with small differences at maximum (Hughes et al., 1982; Chavarren and Calbet, 1999; Zoladz et al., 2000). However, there were significant differences in the linear relationships between V.O_2_-P and HR-P at different cadences. Although both oxygen uptake and heart rate increased at 120 rpm compared to 60 rpm and 90 rpm, the changes in V.O_2_ and HR per watt, as indicated by the slopes, were relatively lower at 120 rpm than at 60 rpm and 90 rpm. The higher absolute values at equivalent intensities can be attributed to the higher baseline levels, probably indicating greater metabolic cost associated with leg movement (i.e., internal work) at higher pedaling rates (Francescato et al., 1995).

In line with previous observations, blood lactate concentration increased exponentially with work rate, irrespective of pedaling rate (Zoladz et al., 2000). While blood lactate concentration converged with increasing intensity at 60 rpm and 90 rpm, this was not observed for a cadence of 120 rpm. The baseline levels, time constants, and amplitudes associated with the pedaling rate of 120 rpm led to higher starting levels, a faster rise in blood lactate concentration with increasing work rate, and a higher maximum concentration. These findings may indicate an increased reliance on glycolytic pathways and anaerobic processes at 120 rpm cadence, suggesting a different energy production composition compared to the other pedaling rates (Beneke et al., 2018). This pattern is also evident in the higher metabolic costs associated with 120 rpm, independent of work rate. In agreement with previous research, pedaling rate significantly influences the metabolic cost of cycling, with lower cadences demonstrating greater bioenergetic efficiency (Hagberg et al., 1981; Böning et al., 1984; Sargeant, 1994; Foss and Hallén, 2004).

The observed physiological differences between pedaling rates can be attributed to biomechanical and muscular factors. At a cadence of 120 rpm, the higher rate requires a more rapid cycle of muscle contraction and relaxation, potentially leading to an increased reliance on predominant glycolytic fast-twitch muscle fibers, which are designed for rapid and powerful contractions. These fibers are efficient for fast movements but have a lower oxidative capacity compared to slow-twitch fibers, which may result in lower oxygen demand per watt, and subsequently a reduced heart rate per watt. This could explain why, despite the faster pedaling rate, both oxygen uptake and heart rate per watt were lower at 120 rpm compared to 60 rpm and 90 rpm.

### 4.2 Changes in optimal pedaling rate with increasing work rate

The optimal pedaling rate in cycling is not a fixed value, but varies depending on the individual’s muscle fiber type distribution, exercise intensity, duration and fatigue (Dunst et al., 2021). Previous studies have reported different optimal pedaling rates, ranging from 30–60 rpm (Eckermann and Millahn, 1967; Hess and Seusing, 1963; Michielli and Stricevic 1977) to 80–100 rpm for endurance cycling (McKay and Banister, 1976; Hagberg et al., 1981). These differences may be attributed to the data points analyzed in these studies - focusing on specific aspects of endurance performance - and the modeling techniques used. The only study which evaluated systematic changes in optimal pedaling rate with intensity found a linear rise in optimal pedaling rate with increasing power output (Coast and Welch, 1985). Linear models based on submaximal data may provide a reasonable approximation, but a sigmoidal relationship becomes apparent when interpolation and extrapolation techniques are applied. Our study identified a systematic sigmoidal increase in optimal pedaling rate with increasing work rate, from approximately 45 rpm at minimal intensity to approximately 85 rpm at maximal aerobic effort for heart rate, oxygen uptake, and blood lactate concentration. This finding is consistent with previous research results indicating that the most efficient pedaling rate is within the range of 40–85 rpm (Hagberg et al., 1981; Sargeant, 1994; Foss and Hallén, 2004). Interestingly, some studies report a preference of a higher cadence (90–105 rpm) during long and intense efforts, regardless of the performance level (Marsh and Martin, 1997). This preference has been attributed to a reduction of neuromuscular fatigue due to lower muscle force (Sarre and Lepers, 2005). However, the reason why cyclists choose a cadence with lower mechanical efficiency instead of the most economical one remains unknown (Buśko, 2004).

### 4.3 Optimal pedaling rate at characteristic metabolic states

A significant increase in optimal cadence was observed at characteristic metabolic states. Our findings suggest that the optimal utilization of oxidative metabolism, without substantial glycolytic activity (as indicated by LT1), occurs at approximately 65 rpm. This observation is consistent with previous research by Buśko, (2004), which found that maintaining a cadence of 60 rpm during submaximal intensity exercise resulted in the lowest blood lactate concentration, as well as favorable pH, pCO_2_, and HCO_3_ levels, compared to cadences ranging from 40 to 100 rpm.

Contrary to the assumptions of Abbiss and others (2009) and Beneke et al. (2018), the optimal cadence did not show significant inter-individual differences across different characteristic metabolic states, implying a subordinate influence of an athlete’s muscle fiber type distribution and physiological performance level (i.e., V.O_2max_, P_max_). This is in line with the results of Leo et al. (2022), which showed that the variations in maximum average power between power levels were related to torque and did not show significant differences in cadence. Instead, these systematic changes may reflect changes in the recruitment pattern of the propulsive muscles due to the different properties of the different types of muscle fibers. According to Henneman’s size principle (Henneman and Mendell, 1981), the force demand may dictate the specific recruitment pattern of muscles. This concept has faced controversy as it does not account for the recruitment of fast-twitch muscle fibers at very low resistances or forces (Hug et al., 2022). Our results suggest that it is necessary to consider both force and velocity dimensions in recruitment patterns.

The observed differences in the physiological responses across the three different pedaling rates reflect an increasing energy demand at higher pedaling rates, which may be caused by an extensive recruitment of bioenergetically less efficient fast-twitch fibers (Böning, 2017). Our results suggest that each metabolic threshold represents a recruitment pattern changing point at which a higher motor unit begins to contribute to power output, and that also on a macroscopic level each muscle fiber type has a maximum F/v and P/v relationship beyond which no further propulsive power can be generated without recruitment of faster twitching muscle fibers. This assumption is supported by the tri-exponential increase in optimal cadence with intensity for blood lactate concentration, whose fast and slow components indicate at least two different muscle fiber types with specific optimal and maximal movement velocities being recruited during intense endurance exercise. Considering the sigmoidal alteration in optimal pedaling rate concerning blood lactate concentration, slow-twitch oxidative type I fibers exhibit an optimal pedaling rate of approximately 65 rpm, coinciding with the highest intensity without significant glycolytic activity. With further increasing work rate, predominantly glycolyticly working type IIa fibers are recruited, leading to an increase in glycolytic activity until the maximal aerobic power is reached, with a corresponding optimal pedaling rate of 85 rpm. This observation aligns with previous research suggesting a similar optimal rate during peak oxygen uptake (Michaeli set al., 1942) or maximal intensive endurance exercise (Foss and Hallén, 2004). The presence of the second fast component (referred to as “medium component” or “MC” in Figure 6) in the PR_opt_-BLC relationship suggests a possible corresponding recruitment of hybrid fibers. These hybrid fibers can exhibit morphological characteristics of both slow-twitch type I and fast-twitch type IIa muscle fiber types. The existence of hybrid fibers is influenced by factors such as training condition and genetics (Plotkin et al., 2021).

**FIGURE 8:**
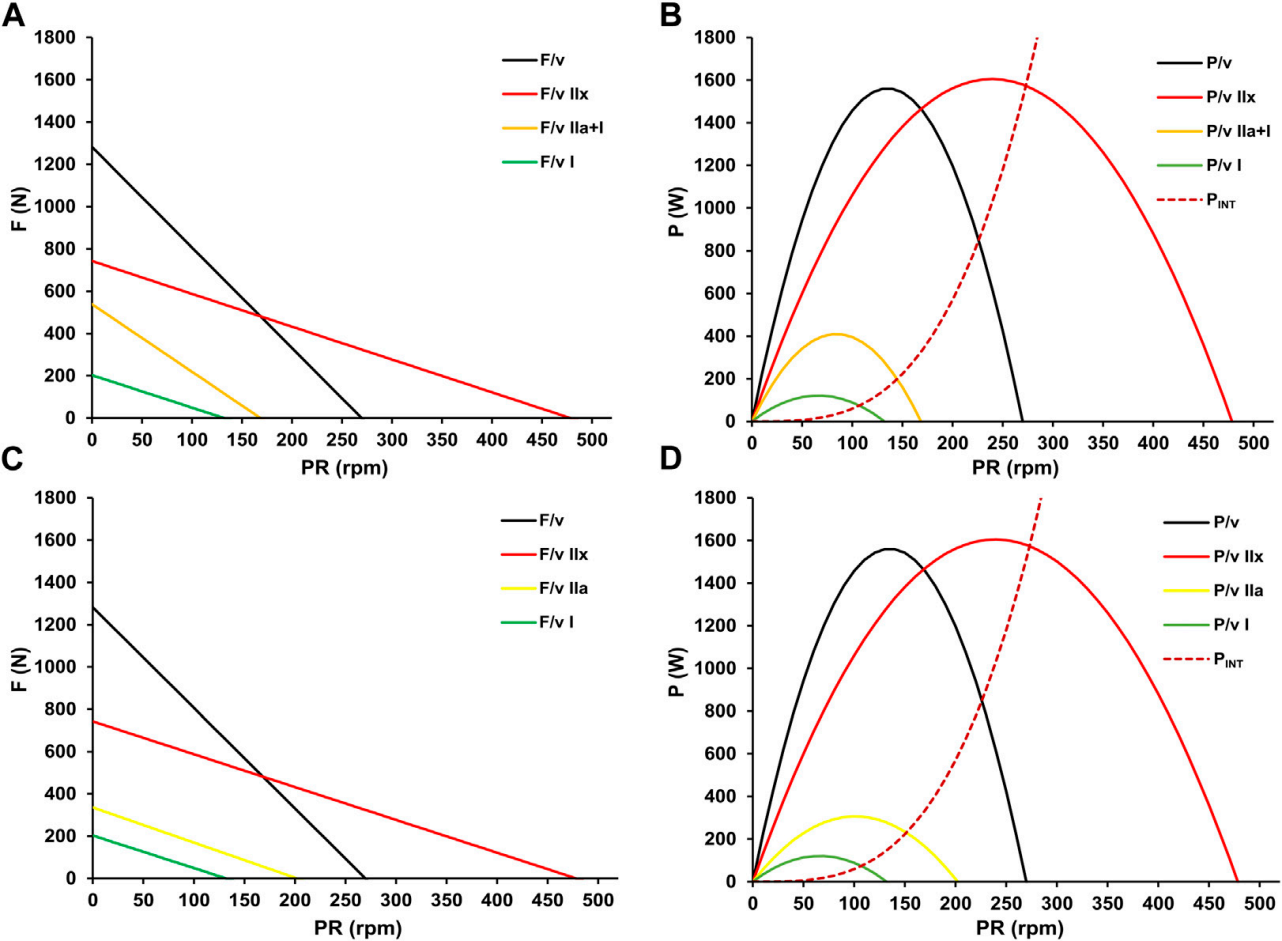
Fiber-type specific (mean pedal) force-velocity **(A)** and power-velocity relationships **(B)** with their cumulative maximal propulsive F/v and P/v profiles (black). The red solid profiles, interpreted as the maximal profiles of fast-twitch glycolytic fibers (F/v IIx, P/v IIx), are obtained by subtracting the *y*-axis intercepts of the orange F/v profile at V.O_2max_ from the fatigue-free maximum (black). Since power output at V.O_2max_ indicates the threshold beyond which further increases in power output do not correspond to an increase in oxygen uptake, the orange profiles (F/v I + IIa, P/v I + IIa) are interpreted as common profiles of slow-twitch oxidative and fast-twitch oxidative muscle fibers. The yellow profiles (F/v IIa, P/v IIa) represent the maximal profiles of fast-twitch oxidative fibers and are derived by subtracting the *y*-axis intercepts of the green F/v profile at LT1 from that of the orange F/v relationship. The LT1 level is interpreted as the maximal power output without significant glycolytic activity, thus representing the profiles of slow-twitch oxidative fibers (F/v I, P/v I). The red dotted line estimates the increase in non-propulsive internal energy expenditure for segmental movement with increasing pedaling rate (P_INT_), as outlined by Minetti (2001).

Consequently, motor units are activated to meet the energy demand (Beltman et al., 2003), but the actual power output depends on the chosen pedaling rate (Dunst et al., 2023b). Therefore, selecting a cadence based on intensity-specific optimization is strategically important as it augments power reserves, potentially extending work duration at a given intensity or increasing power output for a given duration. Incorporating the fatigue-free F/v and P/v relationship into the approach allows for the display of the entire F/v and P/v continuum. This enables the calculation of fiber-type specific profiles on a macroscopic level by subtracting the surfaces of the different profiles, as shown in Figure 8.

According to our calculations, the theoretical maximum pedaling rate of fast-twitch glycolytic IIx fibers is approximately four times higher than that of slow-twitch fibers. Additionally, the maximum power of these fibers exceeds that of fast-twitch Type IIa fibers by five times and that of slow-twitch Type I fibers by more than twelve times. These results are consistent with previous (*in vitro*) observations on the properties of specific fiber types (Hill, 1922; Tesch et al., 1989; Macintosh et al., 2000; Bottinelli et al., 1999; Plomgaard et al., 2006). However, beyond a certain pedaling rate, energy costs for internal work associated with the movement of the lower limbs increase (Francescato et al., 1995). This impairs the conversion of the muscles’ power potential into propulsive mechanical power output. Minetti (2001) proposed a formula to calculate internal force expenditure (P_INT_) based on pedal revolution velocity and body weight. The red dashed line in Figure 8 was calculated applying this formula to our data.

Based on our model, the observed differences in metabolic response at equal work rate for different pedaling rates may be attributed to the specific contributions of different muscle fiber types and associated energy metabolic pathways at specific power output-pedaling rate conditions. This assumption is supported by our recent results demonstrating a dependence of the amount of blood lactate accumulation during a short maximal cycling sprint on pedaling rate (Haase et al., 2024). To accurately determine the maximal performance capacity of different energy metabolic pathways, it seems necessary to test at the respective optimal movement velocity.

In contrast to our findings, Beneke et al. (2016) reported an independence of power output at MLSS from cadence, contradicting previous findings by Denadai et al. (2006). Upon closer examination of Beneke’s data, the authors analyzed the power output and corresponding metabolic state at MLSS for both 60 rpm and 105 rpm and finally concluded that, despite the significantly higher blood lactate concentration at 105 rpm, the power output corresponding to MLSS remained constant, indicating an independence from cadence. In our study, the mean optimal cadence at MLSS was approximately 82.5 rpm. Therefore, in the study by Beneke et al. (2016), both pedaling rates used could have been equidistant from the actual optimum. Calculations using our data for power output at 60 rpm and 105 rpm at MLSS yield 255 W for both cadences. However, applying our model to the cadence studied by Denadai et al. (2006) (50 rpm and 100 rpm) results in a 30 W difference. This discrepancy corresponds to a deviation of more than 10% from the maximal power of 273 W. Consequently, the different results of Denadai et al. (2006) and Beneke et al. (2016) may be attributed to the chosen cadences.

The approach presented here can explain previously unexplained differences in performance diagnostic results. Research has demonstrated that even with equivalent mechanical power demands, treadmill incline can impact running test results (Jones and Doust, 1996). Furthermore, in many studies, particularly outside of cycling, significant linear relationships were not found between heart rate and power output, and between oxygen uptake and power output. Both phenomena could be attributed to movement velocity-specific patterns of fiber recruitment, as proposed by our presented model.

Although the results suggest a clear dependence of power output at different metabolic thresholds on pedaling rate, the question of its significance for training effect remains unanswered. It is conceivable that training on the same power output parabola may lead to similar or vastly different adaptation effects. Further investigations are necessary to examine the training effect of pedaling rate more closely.

### 4.4 Practical applications

The findings highlight the importance of selecting an appropriate cadence in cycling, as the optimal pedaling rate varies significantly depending on the exercise intensity. These particular pedaling rates can improve power output at a given metabolic rate, resulting in better performance and reduced fatigue over time. The small variations in intensity-specific optima among individuals indicate that these empirical values can serve as a useful reference for exercise programming. Additionally, our findings highlight the significance of integrating the dimension of movement velocity into Henneman’s hierarchical size principle. As a result, intensity zones should be presented as a function of movement velocity rather than in absolute terms. Moreover, there is a need to expand the current critical power model to incorporate movement velocity. Taking into account movement velocity will provide more accurate training and pacing recommendations, as heart rate, oxygen uptake, and blood lactate concentration vary depending on cadence at the same work rate, making them inadequate as the only control parameters. The proposed differentiation will potentially offer new perspectives for enhancing the performance capacity of various types of muscle fibers through training.

### 4.5 Limitations

The model presented in this study equates velocity to the crank angular velocity. Our results are therefore interpreted from a macroscopic perspective, considering the propulsive muscle mass as a coherent unit. Although different muscles with specific fiber type distributions contribute during the various phases of a pedal stroke, with specific changes occurring with increasing pedaling rate (So et al., 2005), we observed systematic changes in cardiopulmonary and metabolic responses at all work rates up to maximal aerobic effort. Consequently, the calculated F/v and P/v profiles appear to predominantly reflect the muscle with the lowest aerobic capacity and the highest glycolytic power, requiring the recruitment of larger motor units at a given work rate. This idea is supported by the research of Wang et al. (2020), according to which the contribution of the vastus lateralis muscle, which has the highest proportion of fast-twitch glycolytic fibers among the propulsive muscles in cycling, significantly determines the performance of elite cyclists.

Moreover, the study involves considerable mathematical modeling of physiological responses during maximal exercise. It is important to note that while the individual models are of high quality, this approach simplifies interrelationships and systematic dependencies. Additionally, it must be recognized that the quality of the models decreases as extrapolation extent increases. Therefore, caution is necessary when extending predictions beyond the available data. To determine the parameters for the model functions, we calculated interpolated grid points that were adjusted according to the individual minima, maxima, and parameter curves. However, it should be noted that the selection of grid points may slightly affect the solution.

The heart rate-power relationship was modeled using linear functions, excluding the high-intensity test data. Despite this exclusion, the results obtained from these linear functions remained consistent and closely matched the cadence-specific patterns observed for other parameters. However, the outliers indicated an earlier deviation of heart rate from the linear function to form a leveling-off than for oxygen uptake. This difference may be due to cardiopulmonary factors, which could be linked to an exaggerated increase in cardiac output per heartbeat at frequency limits, ensuring adequate oxygen transport even at high-intensity levels. This limitation can be resolved by utilizing e-functions, representing the levelling-off of ventilation when reaching peak oxygen uptake that can be accompanied by an increase in stroke volume per heartbeat.

Finally, the study has some methodological limitations that had to be accepted for reasons of test feasibility. Firstly, the adequacy of the 3-min period to achieve a true steady state during the incremental test remains debatable, particularly in terms of blood lactate concentration. Secondly, performing all the tests on a single day may have influenced the performance results, especially for the high intensity tests. Although the model descriptions suggest the mathematical plausibility of the data, this may have compromised the validity of the results. Additionally, the non-randomized cadence setting during the incremental test may have introduced an order effect. To address these limitations, future studies should use a more robust experimental design and incorporate direct muscle analysis methods such as biopsy or high-density EMG to increase the validity and interpretability of the results. However, invasive or restrictive measures were not considered viable for the national elite cohort included in this study.

### 4.6 Conclusion

In summary, our study reveals a consistent and systematic increase in the optimal cycling cadence with intensity, following a sigmoidal pattern. This increment spans from 45 rpm during very low-intensity efforts to 85 rpm at maximal aerobic effort, irrespective of the specific physiological parameter considered. At characteristic metabolic states, a significant increase in the optimal cadence was observed, with PR_opt_ increasing from 65 rpm at LT1 to 85 rpm at V.O_2max_, displaying no significant inter-individual variations. State specific changing points in optimal pedaling rate suggest an increased recruitment of faster muscle fiber types with increasing intensity and velocity. The previously postulated two-dimensional metabolic profiles and training zones lack accuracy, as they neglect the crucial dimension of movement velocity. Our findings provide valuable insights into how cyclists can optimize their performance by adjusting their cadence in response to varying work rates and metabolic states. This may potentially lead to increased cycling performance.

## Figures and Tables

**Table:** 

**a**	Slope of the F/v relationship
**b**	*y*-axis intercept of the F/v relationship
**BLC**	Blood lactate concentration
**BLC_i_^-1^(P)**	Relationship between blood lactate concentration and power output at cadence i
**BLC_max_**	Post-exercise maximum blood lactate concentration
**bpm**	Heartbeats per minute, nit of heart rate
**CE**	Caloric equivalent
**C_EHRi_**	Cycling efficiency for heart rate
**C_EV.__O2i_**	Cycling efficiency for oxygen uptake
**F**	Force, mean pedal force
**FC**	Fast component
**F_max_**	Theoretical maximum mean pedal force
**FAT_max_**	Work rate associated with the peak fat oxidation
**F/v**	Force-velocity relationship
**h**	Hour
**HCO_3_**	Bicarbonate
**HI**	High-intensity, power output at or above 90% of power output at maximum oxygen uptake
**HR**	Heart rate
**HR_Base_**	Baseline level of heart rate
**HR_i_^−1^(P)**	Relationship between heart rate and power output at cadence i
**HR_ij_**	Cadence- and workrate-dependent heart rate
**Hz**	Hertz, unit of frequency
**i**	Pedaling rate: i ∈ {60; 90; 120}
**kg**	Kilogram, unit of mass
**L**	Litre
**LT1**	First lactate threshold, highest work rate without significant blood lactate accumulation
**j**	Work rate: j ∈ {100;….; PV.O_2max_}
**m**	Meter, unit of distance
**M**	Mean value
**η^2^**	Eta squared
**MC**	Medium component
**min**	Minute
**ml**	Milliliter
**µl**	Microliter
**MLSS**	Maximum lactate steady state
**mmol l^-1^**	Millimole per liter of whole blood
**N**	Newton, unit of force
**P/v**	Power-velocity relationship
**P_max_**	Maximum power output
**P_peak_**	Highest measured power output
**pCO_2_**	Partial pressure of carbon dioxide
**pH**	Potential of hydrogen, unit of acidity
**P_INT_**	Internal power expenditure
**PR_i_**	Specific pedaling rate, where the index indicates the referred value
**PR_max_**	Maximum cadence, maximum pedaling rate
**PR_opt_**	Cadence at maximal power output, optimal pedaling rate
**PV.O_2max_**	Power output at maximum oxygen uptake
**r**	Crank length
***R* ^2^**	Coefficient of correlation
**RER**	Respiratory exchange ratio
**RER_ij_**	Cadence- and workrate-dependent respiratory exchange ratio
**rpm**	Crank revolutions per minute
**s**	Second
**SC**	Slow component
**SD**	Standard deviation
**τ**	Tau, time constant of kinetic
**VE**	Minute ventilation
**V.E·VO_2_^−1^**	Minute ventilation to oxygen uptake ratio
**v.La_max_**	Maximal rate of lactate accumulation
**V.O_2_**	Oxygen uptake
**V.O_2Base_**	Baseline level of oxygen uptake
**V.O_2max_**	Test specific maximal oxygen uptake
**V.O_2ij_**	Cadence- and work rate-dependent oxygen uptake
**V.O_2i_^−1^(P)**	Relationship between oxygen uptake and power output at cadence i
**W**	Watt, unit of power
**W kg^-1^**	Relative power output, watt per kilogram of bodyweight
**W_MET_**	Metabolic cost
**Δ_BLC_**	Blood lactate accumulation

## Data Availability

The raw data supporting the conclusions of this article will be made available by the authors, without undue reservation.

## Footnotes

1 See chapter 2.7.
