# Supplementary material for: Understanding optimal cadence dynamics: a systematic analysis of the power-velocity relationship in track cyclists with increasing exercise intensity
Source: Front Physiol. 2024 Apr 5;15:1343601. doi: 10.3389/fphys.2024.1343601 (PMC11027132; doi:10.3389/fphys.2024.1343601)
Supplement: Supplementary file 1 [file DataSheet1.docx]

**Appendix**

**A I:** Calculation of the maximal lactate accumulation rate

The maximal rate of test-specific blood lactate accumulation (v̇La_max_) was calculated using the formula:

${\dot{\text{v}}\text{La}}_{\text{max}}\text{= }\frac{\text{∆BLC}}{\text{T-}\text{t}_{\text{alac}}}\text{ }$ (A1)

where ΔBLC is the blood lactate accumulation during the sprint, calculated as BLC_max_−BLC(0). Here, BLC_max_ represents the maximal post-exercise blood lactate concentration, and BLC(0) represents the pre-start level. T represents the sprint duration, and t_alac_ is the initial timespan without significant glycolytic activity, determined as the first systematic deviation from the fatigue-free F/v profile, as detailed in our recent publication (Dunst et al., 2023). The concentration of blood lactate after exercise was analyzed using non-linear regression with the 3-parameter model recommended by Beneke et al. (2005) to calculate BLC_max_:

$\text{BLC}\left( \text{t} \right)\text{=}\frac{\text{A∙}\text{k}_{\text{1}}}{\text{k}_{\text{1}}\text{-}\text{k}_{\text{2}}}\text{∙}\left( \text{e}^{\text{-}\text{k}_{\text{1}}\text{∙t}}\text{-}\text{e}^{\text{-}\text{k}_{\text{2}}\text{∙t}} \right)\text{+BLC(0)}$ (A2)

with A denoting the extra-vascular increase and k_1_ and k_2_ the rate constants for the accumulation and subsequent reduction in blood lactate, respectively.

**A II:** Calculation of the different metabolic thresholds

Metabolic thresholds, including lactate threshold 1 (LT1), defined as the highest work rate without significant blood lactate accumulation, peak fat oxidation (FAT_max_, synonymous with Mader's maximum pyruvate deficit), and maximal lactate steady state (MLSS, the point where lactate formation equals the maximal rate of lactate elimination via oxidative phosphorylation), are determined using Mader's approach (1984; 2003). This approach models oxidative phosphorylation and glycolysis as functions of cytosolic phosphorylation state and muscle cell power output.

To identify these thresholds, knowledge of the intensity-specific steady-state activity of oxidative phosphorylation (V̇O_2ss_) and glycolysis (v̇La_ss_) is essential (Mader, 1984; Mader & Heck, 1986; Mader, 2003). Both V̇O_2ss_ and v̇La_ss_ can be expressed using specific Michalis-Menten equations, which involve the activation of a single enzyme dependent on substrate concentration and the maximal performance of oxidative phosphorylation and glycolysis.

The intensity-specific activity of V̇O_2ss_ can be expressed via the following equation knowing the exercise-specific maximal oxygen uptake (V̇O_2max_) (Mader & Heck, 1986; Mader, 2003):

$\dot{\text{V}}\text{O}_{\text{2SS}}\text{=}\frac{\dot{\text{V}}\text{O}_{\text{2max}}}{\text{1+}\frac{\text{ks1}}{\text{[ADP}\text{]}^{\text{2}}}}$ (A3)

where the ADP concentration indicates the current energy demand and ks1=0.0635 reflects the 50% activation constant of oxidative phosphorylation.

The intensity-specific activity of v̇La_ss_ can be expressed via the following equation knowing the exercise-specific maximal lactate accumulation rate (v̇La_max_) (Mader & Heck, 1986; Mader, 2003):

$\dot{\text{v}}\text{La}_{\text{SS}}\text{=}\frac{\dot{\text{v}}\text{La}_{\text{max}}}{\text{1+}\frac{\text{ks2}}{\text{[ADP}\text{]}^{\text{3}}}}$ (A4)

where the ADP concentration indicates the current energy demand and ks2=(1.1)^3^ reflects the 50% activation constant of glycolysis.

The unknown ADP concentration can be substituted using Equation (A2) and (A3) as follows:

$\left( \frac{\text{ks1∙}\dot{\text{V}}\text{O}_{\text{2ss}}}{\dot{\text{V}}\text{O}_{\text{2max}}\text{-}\dot{\text{V}}\text{O}_{\text{2ss}}} \right)^{\frac{\text{1}}{\text{2}}}\text{=}\left[ \text{ADP} \right]\text{=}\left( \frac{\text{ks2∙}\dot{\text{v}}\text{La}_{\text{ss}}}{\dot{\text{v}}\text{La}_{\text{max}}\text{-}\dot{\text{v}}\text{La}_{\text{ss}}} \right)^{\frac{\text{1}}{\text{3}}}$ (A5)

Lactate oxidation primarily occurs in active muscles. The rate of lactate oxidation (v̇La_ox_) is a linear function of the rate of oxygen consumption. This relationship is influenced by the amount of pyruvate/lactate oxidized per unit of oxygen and the distribution volume (Mader & Heck, 1986). The rate of lactate elimination was calculated as follows:

$\dot{\text{v}}\text{La}_{\text{ox}}\text{=}\text{CE}_{\text{La.}\text{O}_{\text{2}}}\text{∙}\frac{\dot{\text{V}}\text{O}_{\text{ss}}}{\text{Vol}_{\text{LA}}}$ (A6)

where a lactate-equivalent of CE_La.O2_=0.02049 mmolLa (mlO_2_)^-1^ and a lactate distrubution volume Vol_La_=0.4 were used.

The pyruvate deficit (PD) can be calculated as the difference between the capacity to metabolize pyruvate via oxidative phosphorylation and the pyruvate available due to the current glycolysis rate:

$\text{PD=}{\dot{\text{v}}\text{La}}_{\text{ox}}\text{-}\dot{\text{v}}\text{La}_{\text{ss}}$ (A7)

The term 'maximum pyruvate deficit' denotes the point at which oxidative phosphorylation predominantly utilizes fatty acids for energy production, minimizing the use of pyruvate as a substrate. This indicates the intensity with the highest level of fat combustion, here referred to as FAT_max_:

$\text{FAT}_{\text{max}}\text{=}\max\text{PD}$ (A8)

The MLSS is the point at which the rate of lactate formation equals the maximal rate of lactate elimination via oxidative phosphorylation, calculated as:

$\text{MLSS=}{\dot{\text{v}}\text{La}}_{\text{ox}}\text{-}\dot{\text{v}}\text{La}_{\text{ss}}\text{=0}$ (A9)

Lactate threshold 1 (LT1) corresponds to the point of highest activity of oxidative phosphorylation without significant glycolytic activity. Biochemically, this corresponds to the muscle pH (pHm) value that is relatively highest and can be calculated as follows (Mader, 2003):

$\text{pH}_{\text{m.max}}\text{=}\max\text{(}\text{-}\text{log}\text{(}\text{[H}^{\text{+}}\text{])=}\text{max}\text{(7.85+}\text{d}_{\text{buff}}\text{∙(0.8∙[P}\text{]}_{\text{i}}\text{-[La}\text{]}_{\text{m}}\text{-0.55∙}\text{log}_{\text{10}}\text{(40+55∙}{\dot{\text{V}}\text{O}}_{\text{2SS}}\text{∙}{\dot{\text{V}}\text{O}}_{\text{2max}}^{\text{-1}}\text{)}$ (A10)

where d_buff_ =54 mval l^-1^ pHm^-1^ represents the coefficient equal to the mean buffering capacity of skeletal muscle.

Steady-state oxygen uptake can be assessed using spirometry and described via a linear relationship between V̇O_2ss_ and workload:

$\dot{\text{V}}\text{O}_{\text{2ss}}\text{=}\text{C}_{\text{E}}\text{∙P+}\dot{\text{V}}\text{O}_{\text{2Base}}$ (4.2)

where C_E_ represents the efficiency constants for oxygen uptake and V̇O_2Base_ denotes the baseline oxygen uptake at rest.

Using this equation, the corresponding work rate for any level of V̇O_2ss_ can be calculated:

$\text{P=}\frac{\dot{\text{V}}\text{O}_{\text{2SS}}\text{-}\dot{\text{V}}\text{O}_{\text{2Base}}}{\text{C}_{\text{E}}}$ (A11)

Figure A1 illustrates a representative result of the described procedure.


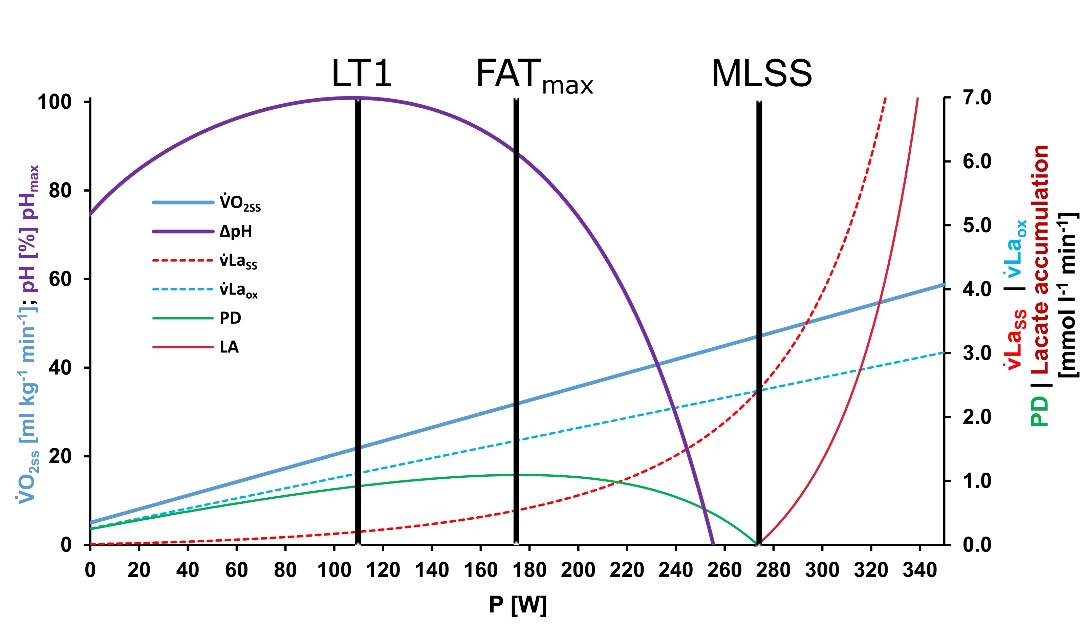


**Figure A1.** The kinetics of steady-state activity of oxygen uptake (V̇O_2ss_), lactate formation (v̇La_ss_), lactate elimination (v̇La_ox_), pyruvate deficit (PD), lactate accumulation (LA) and changes in muscle pH. These calculations are based on the mean values of V̇O_2max_, v̇La_max_ and body weight. The localisation of the thresholds (LT1, FAT_max_, MLSS) is indicated by the vertical black lines.

**References:**

Beneke R, Hütler M, Jung M, Leithäuser RM. Modeling the blood lactate kinetics at maximal short-term exercise conditions in children, adolescents, and adults. J Appl Physiol. 2005;99(2):499-504. doi: 10.1152/japplphysiol.00062.2005.

Dunst, A.K.; Hesse, C.; Feldmann, A.; Holmberg, H.C. A novel approach to determine alactic timespan in the assessment of the maximal lactate accumulation rate in elite track cyclists. Int. J. Sport. Physiol. Perform. 2023, 1, 1–7.

Mader A. Eine Theorie zur Berechnung der Dynamik und des steady state von Phosphorylierungszustand und Stoffwechselaktivität der Muskelzelle als Folge des Energiebedarfs. Köln: Dt. Sporthochschule; 1984.

Mader A, Heck H. A theory of the metabolic origin of "anaerobic threshold". Int J Sports Med 1986, 7(1):45–65.

Mader A, Heck H. Energiestoffwechselregulation, Erweiterungen des theoretischen Konzepts und seiner Begründungen. Nachweis der praktischen Nützlichkeit der Simulation des Energiestoffwechsels. In Brennpunktthema Computersimulation: Möglichkeiten zur Theoriebildung und Ergebnisinterpretation. Edited by Mader A, Allmer H. Sankt Augustin: Academia-Verl; 1996:124–162.

Mader A. Glycolysis and oxidative phosphorylation as a function of cytosolic phosphorylation state and power output of the muscle cell. Eur J Appl Physiol. 2003, 88(4–5):317–338.

Hauser T, Adam J, Schulz H. Comparison of calculated and experimental power in maximal lactate-steady state during cycling. Theor Biol Med Model. 2014;11:25. https://doi.org/10.1186/1742-4682-11-25
